# Case Report: Metreleptin treatment enables successful pregnancy in a female with congenital generalized lipodystrophy

**DOI:** 10.3389/fendo.2025.1715799

**Published:** 2025-11-18

**Authors:** Maria Somali, Emmanouil Korakas, Eirini Pizirtzidou, Zoe A. Efstathiadou, Michail Sapranidis, Zadalla Mouslech

**Affiliations:** 1 Department of Endocrinology, Diabetes and Metabolism, Nexthealth Societé Anonyme (S.A.), General Clinic, Thessaloniki, Greece; 2 Department of Diabetes and Endocrinology, University College London Hospital National Health Service (NHS) Foundation Trust, London, United Kingdom; 3 Department of Nursing, International Hellenic University, Thessaloniki, Greece; 4 Department of Endocrinology , “Hippokration” General Hospital of Thessaloniki, Thessaloniki, Greece

**Keywords:** lipodystrophy, AGPAT2 gene, leptin, metreleptin, fertility

## Abstract

Lipodystrophy is a rare group of conditions characterized by partial or total absence of adipose tissue in the body. The lipodystrophy is either congenital or acquired and is also classified further to either generalized or partial. Lipodystrophy is associated with severe insulin resistance and metabolic disorders due to ectopic fat deposition. The therapeutic approach of lipodystrophy is limited to preventing the exacerbation of complications associated to metabolic disorders and include low fat diet and exercise in combination with medication for diabetes control and fibrates or statins to control dyslipidemia. Metreleptin is an adjunctive injectable treatment for people with generalized lipodystrophy due to leptin deficiency aiming to improve appetite and metabolic markers. Metreleptin treatment has also beneficial impact on liver and kidney function, the cardiovascular and the reproductive system and fertility. In the current paper we present a rare case of a female patient of reproductive age with congenital generalized lipodystrophy type 1 who received metreleptin treatment for the control of her diabetes and metabolic disorders. After 13 months of treatment with metreleptin the patient conceived spontaneously and delivered a healthy neonate. This rare and educational case indicates that metreleptin plays a key role in the adipose-mediated regulation of fertility.

## 1 Introduction

Lipodystrophy is a rare group of conditions characterized by absence (generalized) or abnormal (partial) distribution of adipose tissue in the body (1). Lipodystrophy is either congenital (due to inherited genetic mutations) or acquired (due to autoimmune diseases or medications). Congenital generalized lipodystrophy (CGL) includes different subtypes based on the different gene mutations involved such as *AGPAT2, BSCL2, CAV1, PTRF, LMNA* (e.g., T10I, biallelic lamin A specific variants), *PPARG* (biallelic variants), *PCYT1A, PLAAT3* (2). Acquired Generalized Lipodystrophy (AGL) is caused due to autoimmune disease, panniculitis-associated or is idiopathic. Partial lipodystrophy syndromes are classified into Familial Partial Lipodystrophy (FPLD) including FPLD1 (Köbberling syndrome), FPLD2 (Dunnigan syndrome, due to mutations in the *LMNA* gene), FPLD3 (*PPARG* gene), FPLD4 (*PLIN1* gene), FPLD5 (*CIDEC* gene), FPLD6 (*LIPE* gene) gene mutations. Acquired Partial Lipodystrophy (APL) is associated with autoimmune disorders, membranoproliferative glomerulonephritis (MPGN) or idiopathic causes. Lipodystrophy syndromes are linked to metabolic abnormalities associated with insulin resistance (1, 3). The main cause of insulin resistance in lipodystrophy is the fact that the body stores fat at ectopic sites due to its inability to store energy in the subcutaneous adipose depots. These metabolic defects can lead to chronic complications such as poorly controlled diabetes mellitus, recurrent episodes of acute pancreatitis due to severe hypertriglyceridemia, hepatic cirrhosis, proteinuria and renal failure, ultimately and collectively leading to premature cardiovascular disease. Other clinical manifestations of lipodystrophy are signs of profound insulin resistance such as acanthosis nigricans, polycystic ovarian syndrome (PCOS), and eruptive xanthomas associated with severe hypertriglyceridemia.

Due to extensive fat loss the levels of adipokines i.e. leptin and other hormones derived from the adipose tissue are low (1, 3, 4). Leptin plays an important role in food intake regulation as it regulates appetite, decreases hepatic gluconeogenesis, and improves insulin sensitivity. The therapeutic approach of lipodystrophy was limited, until recently, to low fat diets and exercise in combination with traditional anti-diabetic medications and fibrates or statins to control dyslipidemia. Metreleptin, an analogue of human leptin, is an injectable therapy first approved by the FDA in the USA in 2014, and by EMA in 2018. Metreleptin is indicated as an adjunct to diet as a replacement therapy to treat the complications of leptin deficiency in patients with confirmed congenital or acquired GLD in adults and children 2 years of age and above, and also for patients with confirmed FPLD or acquired PLD over 12 years of age for whom standard treatments have failed to achieve adequate metabolic control. A number of studies in various populations have shown prominent benefits for liver and kidney function, cardiovascular events and reproductive system, enhancing fertility and restoring regular menstrual cycles (4–6). The aim of this article is to present a rare case of a 39-year-old female patient with congenital generalized lipodystrophy type 1 (CGL1) who was initially misdiagnosed with type 1 diabetes mellitus at age 14 (and was later recognized to have severe insulin-resistant diabetes secondary to CGL1), PCOS and irregular menses, who conceived spontaneously after being treated with metreleptin for 13 months prior to conception and delivered a healthy neonate.

## 2 Case presentation

### 2.1 Baseline

A 39-year-old woman presented to our clinic for management of diabetes mellitus. At age 14, she was incorrectly diagnosed with type 1 diabetes mellitus, based solely on her age and clinical presentation, as autoimmune markers and C-peptide were not assessed. Retrospectively, and based on her phenotype and genetic findings, the diabetes was attributed to severe insulin resistance secondary to congenital generalized lipodystrophy. She was initially treated with variable intravenous insulin infusion during hospitalization and was subsequently transitioned to a twice-daily subcutaneous mixed insulin regimen.

Her history revealed that she had been diagnosed with generalized lipodystrophy at the age of 5 months, based on striking clinical features: complete absence of subcutaneous adipose tissue; apparent muscular hypertrophy of both upper and lower extremities; hypotrophy of the gluteal region; abdominal prominence; joint stiffness; and limited hip abduction. Abdominal examination showed hepatomegaly (liver palpable 3–4 cm below the costal margin) and splenomegaly (4–5 cm below the costal margin). Facial features included an appearance older than her chronological age, epicanthal folds, a low nasal bridge, and palpable lymph nodes due to loss of subcutaneous fat. At that time, genetic sequencing was not performed, and therefore the specific causative gene mutation could not be identified or confirmed.

Genetic testing was first performed at the age of 26 years while the patient was under diabetes care in a public hospital diabetes clinic. Molecular analysis of the *AGPAT2* gene, including sequencing of all six coding exons and the intron–exon boundaries by direct sequencing, revealed pathogenic variants associated with congenital generalized lipodystrophy (7). The patient was heterozygous for a nonsense mutation c.202C>T (p.R68X) and a missense mutation c.406G>A (p.G136R), both previously reported in association with congenital generalized lipodystrophy. In addition, she carried a heterozygous variant c.49_51dupCTG (p.L17dup), which has not been described in the medical literature and is therefore of uncertain clinical significance. Parental genetic testing was not performed, as both parents declined analysis; however, given the presence of two pathogenic variants and the classical clinical phenotype, compound heterozygosity is highly probable. At the time of testing, sequencing of other lipodystrophy-associated genes such as *BSCL2, CAV1, PTRF, and LMNA* was not available. A detailed family pedigree showed no other cases of lipodystrophy.

The diagnosis of congenital generalized lipodystrophy type 1 (CGL1) was established based on the combination of characteristic clinical features, biochemical abnormalities, and confirmatory genetic findings. Diagnostic methods included detailed physical examination, metabolic evaluation, and molecular testing of the AGPAT2 gene. Differential diagnoses initially included type 1 diabetes mellitus, due to early-onset insulin-requiring hyperglycemia, and acquired generalized lipodystrophy (AGL), given the absence of prior genetic testing. However, the absence of autoimmune markers, the presence of lifelong fat loss since infancy, and compound heterozygous AGPAT2 mutations confirmed the diagnosis of congenital generalized lipodystrophy. Diagnostic challenges included lack of access to autoantibody assays and genetic testing during childhood, leading to initial misclassification as type 1 diabetes. Prognostically, CGL1 is associated with severe insulin resistance, hepatic steatosis, and hypertriglyceridemia, all of which were present in this patient and guided the decision to initiate metreleptin therapy once available.

The patient presented to our clinic at the age of 30 years, for specialized care due to poorly controlled diabetes mellitus, secondary amenorrhea, hypertriglyceridemia, and hypertension (**Table 1**). At that time, she required a total daily insulin dose of 200 units, administered as a multiple daily injection (MDI) basal–bolus regimen comprising insulin glargine 68 units as basal insulin and insulin aspart 180 units as prandial insulin. She also had a history of polycystic ovary syndrome (PCOS) with secondary amenorrhea. Menarche occurred at 11 years of age. At 18 years, treatment with norethisterone was initiated at a dose of one tablet daily for 7 days every 40 days if spontaneous menstruation did not occur, which successfully induced regular withdrawal bleeding.

**Table 1:** Comparative clinical and laboratory parameters at baseline, 6 months, and 12 months of follow-up.

	Baseline	6 months follow-up	12 months follow-up
BMI (kg/m^2^)	26.7 (height=163cm, body weight=71 kg)	25.6 (height=163cm, body weight=68 kg)	25.6 (height=163cm, body weight=68 kg)
Lipids (mg/dl)	TC=160 HDL-C=41 LDL-C=72 Triglycerides=237	TC=180 HDL-C=38 LDL-C=84 Triglycerides=289	TC=153 HDL-C=52 LDL-C=74 Triglycerides=133
Leptin (ng/ml)			2
Hba1c (%)	8.2	5.4	6.3
TSH (mIU/L)	1,91	–	–
TDD (units/day)	>200	140	77
LFTs (U/L)	SGOT= 28 SGPT=30 γ–GT=19	SGOT= 17 SGPT=19 γ –GT=15	SGOT=15 SGPT=17 γ –GT=14
Adiponectin (μg/ml)	–	2.8	–
Proteinuria	Urine Analysis: normal findings	Urine Analysis: normal findings	Urine Analysis: normal findings UACR (random sample): 16.4 mg/gr

BMI: body mass index body, TC: total cholesterol, HDL-C: high-density lipoprotein cholesterol, LDL-C: low-density lipoprotein cholesterol, HbA1c: glycated hemoglobin, TSH: thyroid-stimulating hormone, TDD: total daily insulin dose, LFTs: liver function tests, SGOT: serum glutamic-oxaloacetic transaminase, SGPT: serum glutamic-pyruvic transaminase, γ-GT: gamma-glutamyl-transferase, UACR: urinary albumin-to-creatinine ratio.

**Figure 1:**
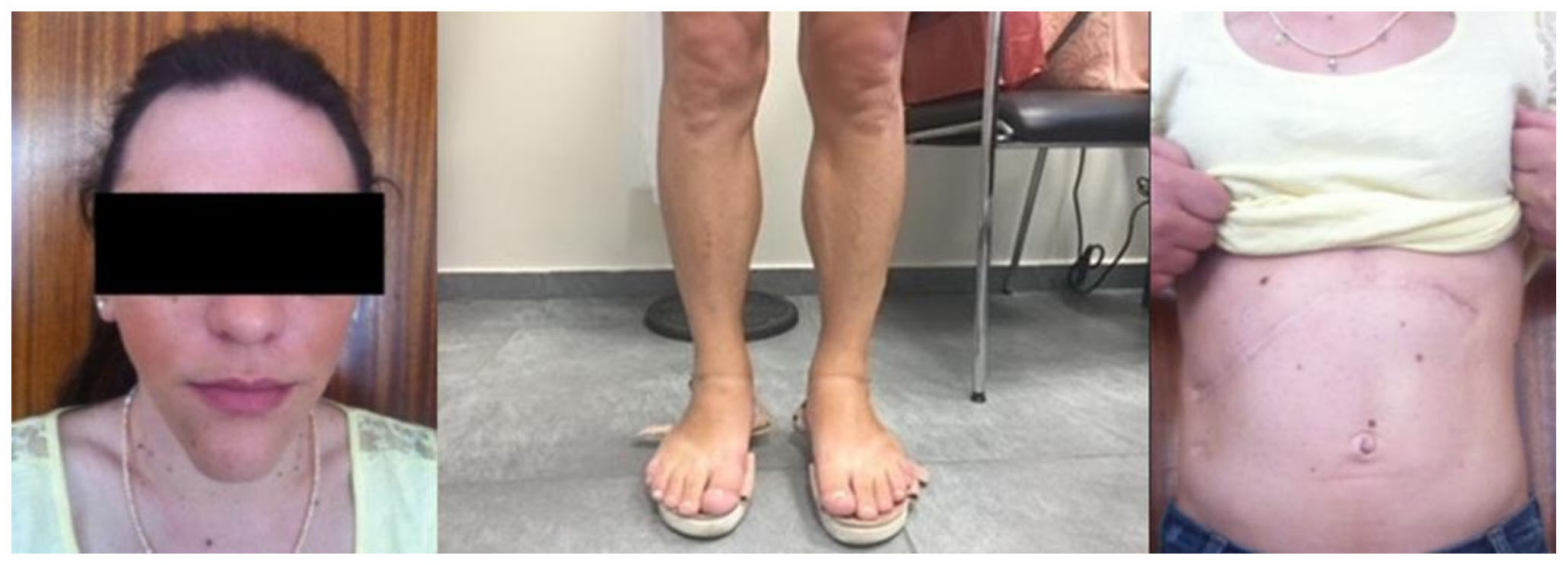
Typical features of generalized lipodystrophy in our patient; appearance older than her chronological age, a low nasal bridge, and palpable lymph nodes due to loss of subcutaneous fat, complete absence of subcutancous adipose tissue, apparent muscular hypertrophy of lower extremities, abdominal prominence.

Clinical examination revealed generalized absence of subcutaneous adipose tissue, marked muscular hypertrophy, and acromegaloid features, including a prominent mandible and enlarged hands and feet, consistent with a CGL1 phenotype (**Figure 1**). Metabolic assessment demonstrated dyslipidemia and low–normal serum leptin levels (**Table 1**). Her past medical history was notable for a right hepatic lobectomy and cholecystectomy in 2005, performed after the incidental discovery of a 10.5 cm hepatic tumor, which histology confirmed as a benign hepatic adenoma. A cardiology evaluation, including echocardiography, revealed no pathological findings. To address severe insulin resistance—a recognized feature of congenital generalized lipodystrophy—we initiated metformin 850 mg twice daily and pioglitazone 15 mg twice daily.

### 2.2 6-months follow-up

At 6-month follow-up, the patient resumed menses, allowing discontinuation of norethisterone, but continued to have oligomenorrhea with ~50-day cycles. Glycemic control improved (HbA1c 6.3%) and total daily insulin fell to ~140 U (glargine 65 U basal; aspart 22–25 U before each meal). Body weight decreased to 68 kg (BMI 25.6 kg/m²). Given her reproductive age, she was referred for genetic counseling. Thyroid and cardiac ultrasounds were normal. Abdominal ultrasound showed a 3.06 cm hypoechoic lesion in the left hepatic lobe, while spleen, pancreas, urinary bladder, uterus, and ovaries were normal in size and echotexture.

### 2.3 12-months follow-up

Liver MRI with and without contrast demonstrated six main nodular lesions and numerous smaller nodules (<1 cm in diameter) scattered throughout the liver parenchyma, with no significant change compared to the previous MRI. Laboratory evaluation showed reduced leptin levels (2 ng/mL; normal range 3.7-11.1 ng/mL) and low adiponectin levels (2.8 µg/mL; normal range 4-20 µg/mL). Levels were measured at a single pre-treatment time point, as repeated assays were unavailable in our setting. In light of these findings and the overall clinical course, pioglitazone therapy was discontinued.

### 2.4 Metreleptin treatment initiation

The patient’s clinical condition remained stable on intensive insulin therapy and regular follow-up, as metreleptin was not yet available in Greece. She was referred to a specialized lipodystrophy center in the United States, but was unable to travel because of financial constraints. Metreleptin (Myalepta) received European Medicines Agency (EMA) approval in July 2018 and became available in Greece shortly thereafter. In May 2020, metreleptin 5.0 mg daily was initiated as causal therapy. The metreleptin dose was selected according to the European Medicines Agency (EMA) Summary of Product Characteristics (SmPC) for Myalepta, which provides weight-based dosing recommendations for generalized lipodystrophy. Based on the patient’s body weight and metabolic profile, an effective dose of 5.0 mg once daily (prepared from the 5.8 mg vial) was appropriate. Over the first 3 months, her total daily insulin requirement decreased to ~80 units (insulin glargine 35 units and insulin aspart 12–15 units three times daily). However, glycemic control subsequently deteriorated, with insulin needs rising again to ~160 units/day and frequent hypoglycemic episodes. To optimize glucose management, a hybrid closed-loop insulin delivery system (Medtronic 780G) was introduced. Before metreleptin initiation, the patient reported increased appetite and frequent snacking between meals. After discontinuation of metreleptin in early pregnancy, appetite remained stable without rebound hyperphagia, likely reflecting compensatory placental leptin production.

### 2.5 6-months after metreleptin initiation

Within weeks of starting metreleptin, she experienced a noticeable reduction in appetite and earlier satiety. After 6 months of treatment with metreleptin, the menstrual cycle was also improved with menses every 38 days. No other type of treatment for induction of the menstrual cycle was used.

### 2.6 13-months after leptin initiation

After 13 months of metreleptin therapy (5.0 mg daily), the patient conceived spontaneously. Metreleptin was discontinued upon confirmation of pregnancy at an estimated 6–8 weeks of gestation, as no clinical safety data or FDA approval exist for metreleptin use during pregnancy. Her diabetes remained well controlled on insulin pump therapy, with HbA1c of 7.0%. Serum leptin levels were not measured during pregnancy, as leptin assays were unavailable in our institution. The pregnancy course was uneventful, and in March 2022, at 38 weeks of gestation, she delivered a healthy male neonate by elective cesarean section. The delivery and postpartum period were free of maternal or neonatal complications. The infant’s birth weight was 3,600 g and length 51 cm; Apgar scores were 8 at 1 minute and 9 at 5 minutes. Respiratory adaptation was normal, and no neonatal hypoglycemia occurred. The only abnormality noted was left-sided cryptorchidism. Metreleptin therapy was re-initiated three months postpartum, after breastfeeding was completed and lactation had ceased.

## 3 Discussion

This rare case highlights the pivotal role of leptin in human reproduction and metabolic homeostasis, while providing real‐world insight into the management of congenital generalized lipodystrophy type 1 (CGL1) with metreleptin therapy. Our patient demonstrated marked metabolic improvement and spontaneous conception after 13 months of metreleptin treatment, ultimately delivering a healthy neonate. To our knowledge, only a single similar case has been described previously, underscoring the novelty and importance of this observation.

Leptin’s role in reproductive function has been well established (8). Leptin indirectly regulates GnRH secretion by acting on specific hypothalamic neurons, as GnRH neurons themselves lack leptin receptors. Its role in the hypothalamus involves both stimulatory and suppressive pathways (9). In addition, it stimulates the translation of GnRHR proteins in gonadotropes, enhancing their sensitivity to GnRH pulses (10). This process is regulated post-transcriptionally by the Musashi protein, which binds to the 3′ untranslated region (UTR) of GnRHR mRNA to repress its translation (11). Leptin promotes the secretion of LH and FSH, which are essential for ovarian follicular development and testicular function, and supports the development of ovarian follicles from the primary to antral stages by amplifying the actions of FSH, LH, and growth factors like IGF-1. It stimulates granulosa and theca cell functions, promoting estrogen and progesterone production, and enhances oocyte maturation in partnership with LH (8, 12).

Congenital generalized lipodystrophy (CGL) is a rare disorder characterized by near-complete absence of adipose tissue, resulting in very low or undetectable circulating leptin levels (1). Affected infants may present with hepatomegaly, umbilical prominence, and hyperphagia due to voracious appetite. As they grow, striking muscular hypertrophy and extensive acanthosis nigricans further accentuate their distinctive phenotype. Females frequently exhibit reproductive abnormalities, including oligomenorrhea, irregular menses, hirsutism, or clitoromegaly, while premature menarche or pubarche are less common (13). Leptin deficiency contributes to anovulation and infertility through two major mechanisms: severe insulin resistance leading to hyperandrogenism and disruption of the normal ultradian (pulsatile) pattern of gonadotropin secretion.

In our patient, menses resumed after initiation of metreleptin, without other hormonal interventions. This finding is consistent with prior reports showing that recombinant leptin improves reproductive function. In a landmark study by Welt et al. (14), administration of recombinant leptin to eight women with hypothalamic amenorrhea increased mean LH concentrations and LH pulse frequency within two weeks. Over three months, follicular development advanced, estradiol levels rose, and three participants achieved ovulatory menstrual cycles. Similarly, Musso et al. (15) treated ten women with CGL using recombinant leptin: eight had amenorrhea at baseline, and all eight regained normal menstrual cycles without changes in ovarian size. These observations suggest that leptin restores normal LH pulsatility, allowing gonadotropins to effectively stimulate ovarian function, while also enhancing insulin sensitivity and lowering serum androgen levels. More recently, Lozno et al. (16) described two sisters with congenital leptin deficiency (CLD)—marked by near-absent leptin but preserved subcutaneous fat—who experienced recovery of regular cycles and normalized GnRH/LH pulsatility following metreleptin therapy. Our patient’s restoration of normal menses with metreleptin parallels these findings, underscoring leptin’s central role in reproductive endocrine regulation and fertility in CGL.

Leptin’s role extends well beyond conception. The human placenta is a significant source of leptin, which acts in autocrine and paracrine fashion to regulate trophoblast proliferation, invasion, and angiogenesis (17). Proper control of these processes is essential for normal placental development. During placentation, subsets of cytotrophoblast and syncytiotrophoblast cells remain attached to the villous basement membrane, whereas others invade the maternal decidua (18). Leptin promotes trophoblast proliferation through mitogen-activated protein kinase (MAPK) signaling, including phosphorylation of MEK and ERK1/2, pathways critical for reproduction, trophoblast invasion, and placental maturation (19). Disrupted leptin signaling has been associated with recurrent miscarriage, pre-eclampsia, gestational diabetes, and intrauterine growth restriction. Women with lipodystrophy are at particularly high risk. In a prospective series of 21 pregnancies in women with generalized or partial lipodystrophy, miscarriage occurred in more than half, and pre-eclampsia complicated half of the live births (20). Among 12 offspring, 1 was small and 3 were large for gestational age, and half experienced neonatal hypoglycemia. Similar findings were reported by Romanisio et al. (21), who followed 48 women with generalized or partial lipodystrophy. Glycemic control was challenging, often requiring insulin doses up to 300 U/day, yet deterioration was uncommon. Our patient’s pregnancy was notable for stability: after metreleptin was stopped at 6–8 weeks for safety concerns, she maintained excellent glycemic control on insulin pump therapy and experienced no hypertensive or obstetric complications, delivering a healthy 3.6-kg infant at term. The maintenance of good glycemic control in our patient despite metreleptin discontinuation likely reflects a combination of factors. First, modern hybrid closed-loop insulin delivery provides automated basal adjustments that markedly reduce glycemic excursions, mitigating the effects of leptin withdrawal. Second, the prolonged metabolic benefits of metreleptin prior to conception may persist beyond treatment cessation, improving hepatic and peripheral insulin sensitivity. Finally, placental leptin production during pregnancy may partially restore circulating leptin levels and contribute to metabolic stability. Unfortunately, serum leptin was not measured during pregnancy, which is a limitation of our report.

Our case shares important features with the only other well-documented report of pregnancy in congenital generalized lipodystrophy treated with leptin, published by Maguire et al. (22) in 2012. Both women had CGL1 due to *AGPAT2* mutations and presented with profound hypoleptinemia, amenorrhea, and severe insulin resistance. In each case, metreleptin therapy normalized gonadotropin secretion and menstrual cycles, leading to spontaneous conception and delivery of a healthy male infant. Nevertheless, several key differences highlight the clinical spectrum of CGL and the flexibility of metreleptin management. The patient described by Maguire et al. began leptin replacement as an adolescent (age 14 years), achieved menarche at 17, and conceived at 23. At conception her HbA1c was 5.7% and she required no insulin, though insulin therapy became necessary from 24 weeks of gestation and escalated to 270 U/day by term. Leptin treatment (0.13 mg/kg/day) was deliberately continued throughout pregnancy to maintain metabolic control. Despite generally good glycemia, the pregnancy was complicated by hydramnios and fetal macrosomia (birth weight 4,200 g), with delivery at 37 weeks complicated by shoulder dystocia and transient neonatal Erb’s palsy. By contrast, our patient started metreleptin much later in life, after more than two decades of poorly controlled diabetes and long-standing amenorrhea. Remarkably, after only 13 months of therapy she resumed regular cycles and conceived spontaneously at 39 years of age. Once pregnancy was confirmed, metreleptin was discontinued at 6–8 weeks’ gestation in view of limited safety data and a clear contraindication in the drug’s SmPC (23), yet metabolic control remained acceptable on continuous subcutaneous insulin infusion (HbA1c 6.5–7.0%). No obstetric complications occurred, and she delivered a healthy 3.6 kg infant by elective cesarean section at 38 weeks. This comparison underscores metreleptin’s unique ability to re-establish reproductive competence in women with CGL, while suggesting that, once conception has occurred and metabolic control is secure, continued treatment may not always be essential for a favorable obstetric outcome.

## 4 Conclusion

This case of a woman with CGL1 who conceived spontaneously after metreleptin therapy provides strong clinical evidence that leptin is indispensable for human fertility. Metreleptin not only improved metabolic parameters but, more importantly, reactivated the hypothalamic–pituitary–gonadal axis, enabling ovulation, conception, and an uncomplicated term pregnancy. As metreleptin becomes more widely used for its licensed indication in generalized lipodystrophy, a multidisciplinary approach among endocrinology, maternal-fetal medicine and reproductive endocrinology specialists will remain crucial to maximize metabolic benefits, ensure maternal and fetal safety, and guide individualized decisions about treatment timing in women who may wish to conceive.

## Data Availability

The original contributions presented in the study are included in the article/supplementary material. Further inquiries can be directed to the corresponding author.
